# Consumer vs. High-End 3D Printers for Guided Implant Surgery—An In Vitro Accuracy Assessment Study of Different 3D Printing Technologies

**DOI:** 10.3390/jcm10214894

**Published:** 2021-10-23

**Authors:** Lukas Wegmüller, Florian Halbeisen, Neha Sharma, Sebastian Kühl, Florian M. Thieringer

**Affiliations:** 1 Medical Additive Manufacturing Research Group (Swiss MAM), Department of Biomedical Engineering, University of Basel, 4123 Allschwil, Switzerland; luke.wegmueller@stud.unibas.ch (L.W.); neha.sharma@usb.ch (N.S.); 2 Basel Institute for Clinical Epidemiology and Biostatistics, Department of Clinical Research, University Hospital Basel, University of Basel, 4031 Basel, Switzerland; floriansamuel.halbeisen@usb.ch; 3 Clinic of Oral and Cranio-Maxillofacial Surgery, University Hospital Basel, 4031 Basel, Switzerland; 4 Department of Oral Surgery, University Center for Dental Medicine, University of Basel, 4058 Basel, Switzerland; sebastian.kuehl@unibas.ch

**Keywords:** three-dimensional, printing, biocompatible materials, computer-aided design, surgical, patient-specific, guided implant surgery

## Abstract

This study evaluates the accuracy of drill guides fabricated in medical-grade, biocompatible materials for static, computer-aided implant surgery (sCAIS). The virtually planned drill guides of ten completed patient cases were printed (*n* = 40) using professional (Material Jetting (MJ)) and consumer-level three-dimensional (3D) printing technologies, namely, Stereolithography (SLA), Fused Filament Fabrication (FFF), and Digital Light Processing (DLP). After printing and post-processing, the drill guides were digitized using an optical scanner. Subsequently, the drill guide’s original (reference) data and the surface scans of the digitized 3D-printed drill guide were superimposed to evaluate their incongruencies. The accuracy of the 3D-printed drill guides was calculated by determining the root mean square (RMS) values. Additionally, cast models of the planned cases were used to check that the drill guides fitted manually. The RMS (mean ± SD) values for the accuracy of 3D-printed drill guides were—MJ (0.09 ± 0.01 mm), SLA (0.12 ± 0.02 mm), FFF (0.18 ± 0.04 mm), and DLP (0.25 ± 0.05 mm). Upon a subjective assessment, all drill guides could be mounted on the cast models without hindrance. The results revealed statistically significant differences (*p* < 0.01) in all except the MJ- and SLA-printed drill guides. Although the measured differences in accuracy were statistically significant, the deviations were negligible from a clinical point of view. Within the limits of this study, we conclude that consumer-level 3D printers can produce surgical guides with a similar accuracy to a high-end, professional 3D printer with reduced costs.

## 1. Introduction

The backward planning in oral implantology combines the precise placement of an implant concerning the available bone volume and the subsequent prosthetic suprastructure. A clinician can place an implant in a pre-defined and predictive manner using static, computer-aided implant surgery (sCAIS) by considering the prosthetic suprastructure [1,2]. For example, the screw channel of the consecutive implant-crown component can be placed in a proper position with respect to the morphology of the replaced tooth and crown. Moreover, sCAIS with drill guides can provide more accurate results than free-hand surgery and reduce the incidence of untoward involvement with neighboring anatomic structures such as the mandibular canal or the maxillary sinus. Furthermore, this technique benefits the patient by allowing flapless surgery, decreasing surgical time, reducing discomfort and swelling, and allowing a quicker return to function. The technique benefits the clinician by reducing chair time, providing the possibility of immediate implant loading, facilitating an accurate means of placing dental implants, and reducing potential surgical complications [1].

To perform sCAIS, implant drill guides, either in milled, compression-molded, or three-dimensional (3D) printed forms, are needed [2]. There are different workflows available for the fabrication of each of these drill guides. The dental technician’s traditional workflow of manufacturing drill guides with compression molding has proven clinically acceptable accuracy. However, this conventional workflow is undoubtedly not without limitations [3]. This workflow contains an initial visit encompassing a conventional impression followed by the fabrication of a stone cast. Subsequently, a dental wax-up and a scan prosthesis, and cone-beam computer tomography (CBCT) are performed to capture the anatomical bone density. After superimposing the scans and planning the implant position, the scan prosthesis must be transformed into a drill guide. This needs at least two patient visits before surgery, and due to more steps in the guide production, a few errors can congregate during the process [4]. Additionally, this entails relatively high production costs.

The continuous improvements in computer-aided design (CAD) and computer-aided manufacturing (CAM) digital workflows have led to the emergence of additive manufacturing (AM), also known as 3D printing technology. This alternative digital workflow consists of only one visit before implant surgery. The workflow consists of a CT or CBCT, and an intraoral scan or the scan of a plaster cast, subsequently aligned in an implant planning software. A virtual wax-up is made with the digital catalog of prosthetic teeth and an implant library containing different implant manufacturers in the planning software. Implants can be “virtually” placed at pre-planned depths and angulations in three dimensions using implant-planning software. Once the digital planning of the implant position is finalized, a drill guide is designed. The CAD file of the drill guide is then exported in a standard tessellation language (STL) file format, which can be either milled or 3D printed. Kernen et al. [4] stated that due to the streamlined and less manual-labor-extensive planning steps using this workflow, the patient benefits from fewer visits and has a fast, precise, safe, and predictable implant surgery with a higher accuracy. Regarding the general accuracy of sCAIS, a horizontal error of approximately 1 mm at the entry point and 1.6 mm at the apex, 0.5 mm in height and with a 5–6° axis, can be found in the literature [5,6].

Compared to consumer-level 3D printers, high-end professional 3D printers are more conducive for producing drill guides made of medical-grade, biocompatible resin materials, as shown in a study by Sommacal et al. [7]. Due to the high initial investment costs of high-end professional 3D printers, it is possible that many clinicians either abstain from purchasing these printers and outsource the drill guide manufacturing, while eventually some clinicians even refrain from using a guided surgical approach altogether. With the evolving technology and improvements in consumer-level desktop 3D printers, along with the availability of several medical-grade, biocompatible, sterilizable materials in the market, there are now several new possibilities in the “chairside-production” of drill guides, enabling clinicians to implement a complete digital workflow in their daily clinical practice.

This study aimed to investigate whether consumer-level desktop 3D printers can produce drill guides for sCAIS with a similar accuracy compared to a high-end, professional 3D printer. The hypothesis was that the consumer-level 3D printers could fabricate drill guides with a similar accuracy to the high-end, professional 3D printer.

## 2. Materials and Methods

Ten patient cases undergoing sCAIS with different implant positions were selected for this study. The drill guides were designed in preoperative implant-planning software (coDiagnostiX^®^, v. 9.0, Dental Wings GmbH, Chemnitz, Germany). The generated drill guide files were then exported in an STL file format for further printing processes. The inclusion criteria were clinical cases requiring dental implants positioned at various anatomical regions and in variable numbers as seen in Table 1. All the guides had a cross-arch design for a maximum stability.

This approach was undertaken to outweigh the potential bias considering the design complexity of the 3D-printed drill guides. The drill guides were then printed with four different 3D printers. The drill guides were fabricated in either Class I or Class IIa medically certified, biocompatible, sterilizable materials [8].

A PolyJet 3D printer (Objet30 Prime, Stratasys Ltd., Minneapolis, MN, USA) based on Material Jetting (MJ) technology was selected in the high-end professional 3D printers category for this study. While in the consumer-level desktop 3D printers category, the following 3D printers were selected—a Stereolithography (SLA) 3D printer (Form 3, Formlabs Inc., Somerville, MA, USA) based on Vat Photopolymerization technology, a Fused Filament Fabrication (FFF) 3D printer (Ultimaker 3 Extended, Ultimaker B.V., Utrecht, The Netherlands) based on Material Extrusion technology, and, lastly, a Digital Light Processing (DLP) 3D printer (Wanhao Duplicator 7 Plus, Wanhao, Jinhua, China) based on Vat Photopolymerization technology. The various 3D printers with their respective printing technologies and material specifications are summarized in Table 2. All of the printers were calibrated according to the manufacturer’s instructions.

### 2.1. Material Jetting (MJ)

In MJ 3D printer (Objet30Prime, Stratasys Ltd., Minneapolis, MN, USA, the drill guides were fabricated with two proprietary photopolymer resins, one as a core biocompatible material (MED610, Stratasys Ltd., Minneapolis, MN, USA), and the other as a water-soluble support material (SUP705, Stratasys Ltd., Minneapolis, MN, USA). The STL files of the drill guides were imported into the 3D printer’s slicing software (Objet Studio Software, v. 9.2.11.6825, Stratasys Ltd., Minneapolis, MN, USA). The drill guides were printed using the automatic placement functionality integrated into the software considering minimal printing time and material consumption. As this technology utilizes water-soluble support structures, the software automatically configures the drill guides angulation for optimal printing results. The drill guides were printed at a layer thickness of 28 microns with a “glossy” surface finish and in a high-speed mode. Post-processing steps were required to remove the water-soluble support structures and were performed in a WaterJet Station (Stratasys Ltd., Minneapolis, MN, USA).

### 2.2. Stereolithography (SLA)

**Figure 1:**
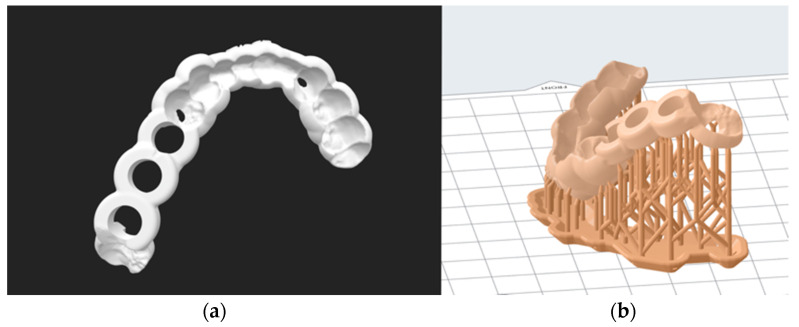
(**a**) STL planning file and (**b**) print preparation (here for the Form 3 using PreForm).

In the SLA 3D printer (Form 3, Formlabs Inc., Somerville, MA, USA), the drill guides were fabricated in a proprietary biocompatible photopolymer resin material (Dental SG Resin, Formlabs Inc., Somerville, MA, USA). The STL files of the drill guides were imported into the 3D printer’s slicing software (PreForm v. 3.4.4, Formlabs Inc., Somerville, MA, USA). Depending upon the complexity of the drill guides, the guides were oriented at a 30°ߝ45° angle generating sufficient raft and support structures (Figure 1). The manufacturer’s recommended settings for the respective biocompatible resin material were selected, and the guides were printed at a layer thickness of 50 microns. After printing, the drill guides were immersed and cleaned with 90% isopropyl alcohol (IPA) solution for 20 min and left to air dry for another 10 min. The drill guides were post-cured for another 30 min in an ultraviolet (UV-A light 385 nm) lightbox following the manufacturer’s instructions. Subsequently, the manual removal of the support structures from the drill guides was performed using fine-cutting pliers.

### 2.3. Fused Filament Fabrication (FFF)

In FFF 3D printer (Ultimaker 3 Extended, Ultimaker B.V., Utrecht, Netherlands), the drill guides were fabricated with two materials, one as a core biocompatible material (Nylon 680, Taulman 680 FDA, Taulman3D LLC, Linton, IN, USA), and the other as a water-soluble support material (ProFill™ PVA, 3D-Printerstore.ch, Weinfelden, Switzerland). The presence of two printheads in this printer enabled the simultaneous usage of these two materials. The STL files of the drill guides were imported into the 3D printer’s compatible slicing software (Cura v.3.6.0 Ultimaker B.V., Utrecht, The Netherlands). The recommended settings for extruder 1 print core AA 0.4 mm nozzle were Generic Nylon, layer thickness 100 microns, and 50% infill. Due to the hygroscopic characteristics of the nylon filament and its accompanying tendency to absorb humidity, the material was dried in a filament dryer (Apium Filament Dryer, Apium Additive Technologies GmbH, Karlsruhe, Germany) before printing and stored in a freezer bag containing silica gel pads. During the printing process, the core material and the water-soluble material were enclosed in a hygroscopic box. Post-processing steps were required to remove the water-soluble support structures and were performed in a WaterJet Station (Stratasys Ltd., Minneapolis, MN, USA).

### 2.4. Digital Light Processing (DLP)

**Figure 2:**
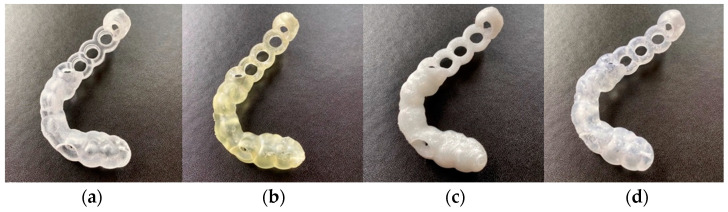
Printed drill guides after removal of support structures: (**a**) Objet30 Prime, (**b**) Form 3, (**c**) Ultimaker 3 Extended, (**d**) Duplicator 7 Plus.

In the DLP 3D printer (Wanhao Duplicator 7 Plus, Wanhao, Jinhua, China), the drill guides were printed with one biocompatible resin material (Freeprint^®^ ortho 405, DETAX GmbH & Co. KG, Ettlingen, Germany). The STL files of the drill guide were imported into the 3D printer’s slicing software (Wanhao D7 Workshop v.1.1.29, Wanhao, Jinhua, China). Similar to SLA 3D printing, the drill guides were oriented at a 30°–45° angle generating sufficient raft and support structures. The manufacturer’s recommended settings for the respective biocompatible resin material were selected, and the drill guides were printed at a layer thickness of 50 microns. After printing, the excess resin was washed with 90% isopropyl alcohol (IPA) solution for 20 min, air dried, and then post-cured for 30 min using a UV-A light 385 nm lightbox. Subsequently, the manual removal of the support structures from the drill guides was performed using fine-cutting pliers. The printed drill guides from one case can be seen in Figure 2.

### 2.5. Subjective Assessment of the Overall Fit of 3D Printed Drill Guides

The drill guides were placed on their corresponding cast models of the preoperative situation to assess the subjective optical and haptic impression of the accuracy of fit.

### 2.6. Digitization Protocol

For the digitization protocol, a hole was drilled on the posterior plane of each drill guide enabling the attachment of a thin metal post. Due to the transparent, shiny surface of the drill guides printed with MJ, SLA, and DLP technologies, a spray powder (ARDROX^®^ 9D1B, Chemetall GmbH, Frankfurt/Main, Germany) for the scanning process was required. Although the FFF 3D-printed drill guides were white, the guide’s surface was overly reflective for the digitization process. Therefore, all the 3D-printed drill guides were coated with a thin layer of spray powder for the digitization process. For the digitization process of the 3D-printed drill guides, surface scans were performed using an optical high-resolution 3D scanner with an accuracy of ≤0.05 mm (EinScan SP, SHINING 3D Tech. Co., Ltd., Hangzhou, China). The point cloud data generated by scanning were then converted and exported into STL format.

### 2.7. Accuracy Assessment

The digitized scans were imported into a medically certified CAD software (Materialise 3-Matic v. 14.0, Materialise, Leuven, Belgium) for accuracy and superimposed with the original planning data. For each drill guide, an *n*-point registration followed by a global registration was performed. The *n*-point registration was completed by selecting three manually placed control points on the original drill guide STL file and the corresponding 3D printed drill guide scan STL file. The selection of these points was performed for each drill guide depending on its respective individual design. Subsequently, the global registration was conducted, ensuring a more accurate match between the original STL file and the 3D printed drill guide scan STL file. Subsequently, the analysis module using the “create part comparison analysis” functionality in the software was used to evaluate the accuracy of the drill guides.

Accuracy was quantified as a trueness measurement representing the deviation between the 3D-printed drill guide and the original STL file. According to ISO 5725-1:1994(en), trueness is “the closeness of agreement between the average value obtained from a large series of test results and an accepted reference value” [9].

The mean positive and negative deviations were recorded for each test group, and the overall deviations were measured using the root mean square (RMS) values. The RMS is the square root of the mean square, which calculates the arithmetic mean of the squared difference between the reference guide and the printed guide and represents the absolute values of the deviations between two different datasets [9]. The measurements were visualized in a color map, illustrating the scale of deviations. This comparison provides a quantifiable value of resemblance following optimum superimposition. The greater the RMS value, the larger the deviation error between the two datasets.

### 2.8. Statistical Analysis

Descriptive statistics mean, standard deviation (SD), median, first- and third-quartile ranges (IQR), and the root mean square (RMS) values were collected for all 3D-printed drill guides from each 3D printer. RMS was calculated for each set to summarize the qualitative characteristics and investigate the accuracy of the drill guides. The Shapiro–Wilk test was applied to verify the distribution of the RMS values for each group of comparison. Then, either one-way analysis of variance (ANOVA) with Tukey–Kramer post hoc pairwise tests or a Kruskal–Wallis test with pairwise Wilcoxon Rank Sum (Mann–Whitney U) post hoc tests, adjusted for multiple-testing using a Holm-Bonferroni correction, was carried out to identify intergroup differences in printing accuracy. All statistical analysis was performed using R statistical software (R v. 4.0.0, The R Foundation for Statistical Computing, Vienna, Austria).

## 3. Results

Regarding the subjective assessment, it was noticed that all 3D-printed drill guides printed with each 3D printer could be positioned without hindrance on their respective clinical cast model. These results showed a first optic and haptic impression of high accuracy for all printers. By quantifying the overall accuracy of the 3D-printed drill guides, the results revealed that the MJ technology had the highest trueness with an RMS (mean ± SD) value of 0.09 ± 0.01 mm and a median (Q1 to Q3) value of 0.09 (0.08 to 0.09) mm, closely followed by SLA-printed drill guides with an RMS (mean ± SD) value of 0.12 ± 0.02 mm and a median (Q1 to Q3) value of 0.12 (0.11 to 0.14) mm. The FFF 3D-printed drill guides revealed an RMS (mean ± SD) value of 0.18 ± 0.04 mm and median (Q1 to Q3) value of 0.17 (0.15 to 0.22) mm, while the DLP 3D-printed drill guides revealed the lowest trueness with an RMS (mean ± SD) value of 0.25 ± 0.05 mm and a median (Q1 to Q3) value of 0.22 (0.21 to 0.27) mm. A summary of all the trueness RMS values for all printers is shown in Table 3.

**Figure 3:**
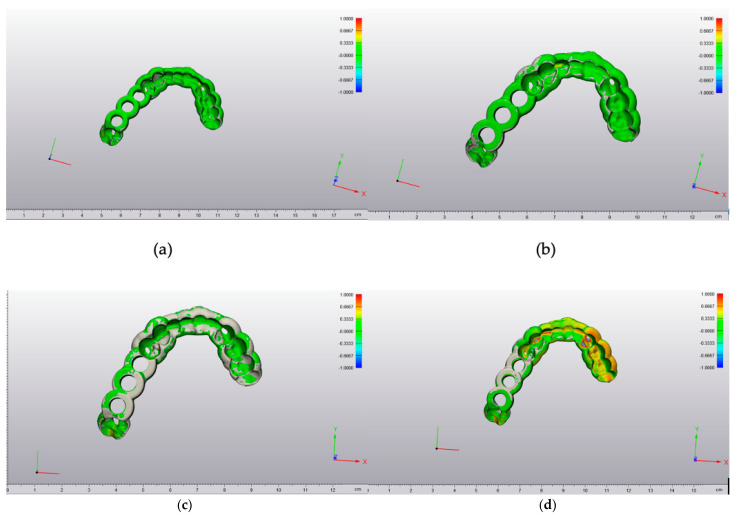
Color-coded deviation maps within each 3D printing technology: (**a**) MJ (**b**) SLA (**c**) FFF (**d**) DLP technology, scale in cm.

The maximum and minimum deviation values were seen from a 3D perspective in a color-coded heat map. A deviation of a particular point in the 3D-printed drill guide can occur in more than one direction. The red-colored regions revealed that the test 3D-printed drill guide was positioned outward with positive deviations. In contrast, the blue-colored regions showed that the test 3D-printed drill guide was positioned inward relative to the original reference STL file with negative deviations (Figure 3).

The results of the other parameters (Table 4), e.g., in the FFF 3D printer, which depicted the lowest mean (0.03 mm) and median (0.02 mm) values compared with the RMS (0.17 mm), also showed that the mean and median values cannot be used to describe accuracy because these values do not consider the variability between the individual measurements.

**Figure 4:**
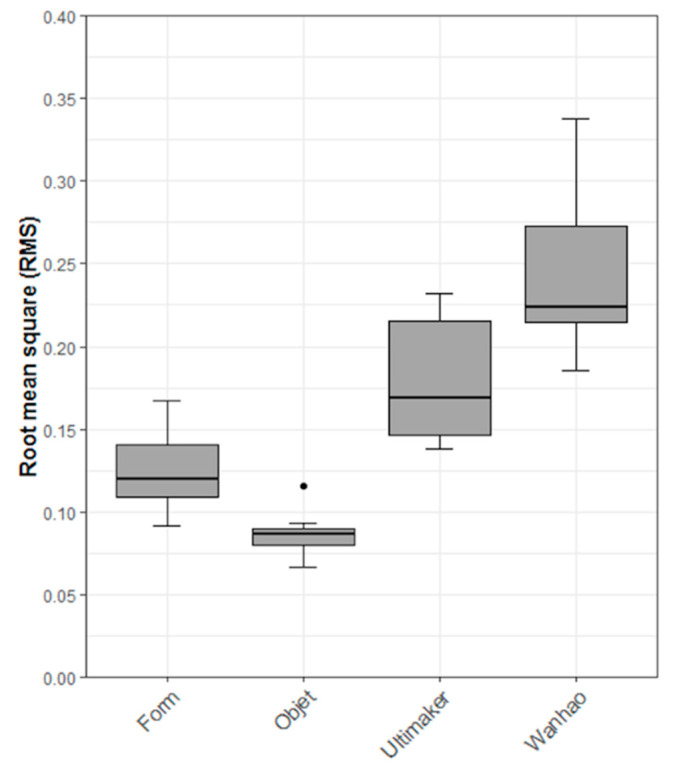
Box plot of the accuracy (trueness) of the root mean square (RMS) values (in mm) for each 3D printer.

The null hypothesis for the Shapiro–Wilk test revealed that the data were normally distributed. Combined with the visual inspection of the Q-Q (Quantile-Quantile) normal plots, we concluded that a parametric statistical test could be used. Based on this, an ANOVA with a Tukey post hoc test was performed. As a sensitivity analysis, a non-parametric Kruskal–Wallis rank-sum test with pairwise Wilcoxon Rank sum tests was performed. The ANOVA showed a statistically significant difference in the accuracy (trueness) of the RMS values between the printers (ANOVA F-value = 40.78, df = 3, *p* < 0.001). The Kruskal–Wallis rank sum test showed a statistically significant difference in the accuracy (trueness) of the RMS values between the 3D printer types (Kruskal–Wallis chi-squared = 31.97, df = 3, *p* < 0.001). The pairwise comparison using the Tukey post hoc test revealed that all comparisons, except the comparison of “SLA—Form 3” to “MJ—Objet,” were statistically significant (Table 5). Combined with Table 3, we conclude that the “Objet30Prime” and “Form 3” printers have the best trueness. The results are also presented graphically as box plots in Figure 4.

## 4. Discussion

The hypothesis was that the consumer-level desktop 3D printers could produce drill guides with a similar accuracy to the high-end professional 3D printer. The present study shows a statistically significant difference in the trueness of the different printing techniques. From the statistic point of view, the null hypothesis has to be rejected. However, from our clinical point of view, the measured scarce differences in the accuracy of the printed drill guides are neglectable. We state this because the drill guides could all be mounted on the associated cast model without hindrance. Additionally, with the possibility of using, for example, an offset of 0.1 mm from the drill guide, as is often recommended from the implant planning software, the deviations are within an acceptable range of error considering that the walls of the drill guide can flex slightly. These results are difficult to compare to previous reports that analyzed the accuracy of 3D-printed surgical guides for sCAIS and studies comparing the accuracy of 3D-printed anatomical bone models [1,10,11,12,13,14,15,16,17,18,19,20,21]. These studies stated an acceptable accuracy for 3D-printed medical devices within their particular field of application, but none used the same method as illustrated in the present study. Most studies analyzed the final implant position for the analysis of implant drill guides, but not simply the guide itself. Juneja et al. [22] and Kim et al. [23] also compared the 3D-printed objects with the digital files and found similar results top those in this study; a mean absolute difference of 0.06 ± 0.05 mm for a PolyJet™ 3D printer. However, these studies examined the discrepancy between the planning and 3D-printed objects based on the mean absolute difference in distance.

Given that deviations in a 3D object can occur in more than one way, one should keep in mind that a mean distance does not depict the actual value of the sum of all deviations. As there are multi-dimensional possibilities of a deviation between the two datasets to occur, the numbers can be both positive and negative. To straighten out the possible bias caused by this, a more accurate number is achieved using the RMS, which results from the square of all values. Subsequently, the software calculates the mean of these square values before taking the square root of the mean value, leading to a more accurate representation of the deviation. An example for this can be seen in Table 2, as the FFF printer shows the lowest mean (0.03 mm) and median (0.02 mm) values compared to the considerably higher RMS (0.17 mm).

There are several possible sources of error regarding the difference between the slightly high maximum/minimum deviations and the low RMS median within one printer series. When operating a 3D printer, one must remember that many modifiable factors can lead to errors in the results. Each printer type comes with its specialties and possible sources of error can conglomerate, as described in a previous study by Msallem et al. [21]. One possible reason for the deviations in the present study could be the need to attach the printed templates to a holder, which were digitally cut out of the scan. Another possibility could be an error in the scanning process as it was difficult for the overhanging parts to be placed at the correct angle for the scanner. If there was an incongruency in the 3D printed template, it would hardly fit the respective cast model, as noticed during the subjective assessment. The accuracy of the 3D scanner is a possible source of error for the measured differences. With the measured accuracies of the printers being larger than the accuracy of the scanner, one can conclude that, in this case, the accuracy of the scanner was sufficient.

Through more overhanging parts and deeper pits, the complexity of the guide did not interfere with the achieved accuracy. One might suggest that, with a more supported guide (for example, the guide fitted on all the available teeth and not just in the neighboring area of the planned implant) comes the possible printing error surface. However, in addition to the literature, we can conclude that a more complex drill guide does not have a less accurate fit [24].

The overall accuracy of sCAIS depends on many factors, where the accuracy of the guide is only one part. It starts with the data acquisition from the CBCT and intraoral scan, and then the data are matched. Furthermore, the design and the accuracy of the drill guide, the fit of the inserted metal sleeve, the intraoperative fit of the guide with or without the mobilizing soft tissues, and the general precision of the surgeon are all considered. Regarding the intraoperative fit, the drill guide must be rigid to prevent bending, and thus compromising the result. According to the manufacturers, the tensile strength differs from 47 MPa to >75 Mpa (Table 1). These numbers give a general impression, but the mechanical properties are dependent on the design of the printed object and should be evaluated separately.

When choosing a 3D printer, one must consider the merits and demerits of each printing technology [25,26]. There are several economic aspects, mostly possible operational pitfalls, and health issues. Economic aspects would be, for example, the size of the build plate, which consequently defines how many guides can be printed simultaneously. Furthermore, the printing speed and the price of the material can make a large difference. The printer types also differ in the usable amount of printing material versus its undeterrable waste during this process (as with FFF printers), the amount used for supports and rafts, or SLA printers’ non-usable resin due to a clouding process in the resin bath.

There are relatively significant differences in how much the printer operator must investigate for the same result regarding operational factors. This begins with the software. There are factory default settings for a specific material provided by the manufacturer’s software; this study used these settings for the MJ, SLA, and FFF printers. On the other hand, there are more open-source variants, such as the software used for the DLP printer. The operator must put in every material-specific parameter, which leaves room for errors and misprints. Moreover, there is also a disparity in the workflow if the support structures are water-soluble, reducing the post-processing time. The need for rinsing off excess resin and then post-curing when using SLA or DLP printers in this study also makes room for possible errors by the operator if the standardized post-processing procedures are not carried out correctly. This is either because not all of the residue is washed off or the object is slightly deformed before the final curing during the transfer from the build plate to the washer and the light-curing box.

When using nylon filaments, it is essential to make sure that the material is dried before use, which is possible either with the method mentioned in the Methods section of this study or also with a drier box. One could use a commercially available light box or a box prepared with holes, a light bulb, and outlining for the filament as described by the manufacturer of the filament on his website (https://taulman3d.com/drying-materials.html, accessed on 15 September 2021). Not doing so would cause problems during printing. The absorbed humidity expands rapidly when heated through the printer’s nozzle, creating air bubbles, and thus inaccuracies and the sticking of nylon to the printhead. Before using the implant drill guides, one must sterilize them, leading to a dimensional change, as seen in a recent study [27,28].

Regarding health, it is crucial to stick to the manufacturer’s published instructions, but the aspect of possible health issues through the exposure to unpolymerized monomers, which are sometimes used in resin-based systems, must be taken into account [29].

The limitations in the findings of this study are the limited number of samples and the lack of the evaluation of the “precision”. Additionally, as this study was a hypothesis-generating study, the statistically significant results need to be addressed in a large confirmatory study. Further studies should establish the standard measurement methods and accuracy minimums for 3D printers for guided implant surgery to become an international norm.

## 5. Conclusions

The study findings show that all the tested 3D printers can print with high accuracy. As expected, the professional MJ 3D printer had the highest trueness (RMS 0.09 ± 0.01 mm), while the DLP 3D printer produced a significantly lower value (RMS 0.25 ± 0.05 mm). Although a statistically significant difference in trueness was observed, all 3D-printed drill guides would be clinically sufficient regarding the overall high accuracy and the possibility to fit them onto cast models for individual oral situations.

The availability of biocompatible 3D printing material, even for consumer-level desktop 3D printers, offers the possibility of using the benefits of 3D-printed, highly accurate products such as surgical drilling and cutting guides, anatomical models for intraoperative pre-bending, or the visualization with a lower financial entry gate than with a high-end professional 3D printer.

With rising healthcare costs, it is pleasing for a clinician not to accumulate more costs when using the newer, more beneficial technology. This study showed that it was possible to print drill guides with an acceptable accuracy using economical, consumer-level desktop 3D printers as opposed to a high-end professional 3D printer.

## Figures and Tables

**Table 1:** Implant case details.

Case	Implant Site (FDI)	Support	Kennedy Class
1	11, 21	tooth	IV
2	11	tooth	III
3	11	tooth	III
4	11	tooth	III
5	25, 26	tooth/tissue	II
6	45, 47	tooth/tissue	II
7	35, 36	tooth/tissue	II
8	24, 25	tooth	III
9	45, 46, 47	tooth	III
10	14, 15	tooth/tissue	III

**Table 2:** 3D printers, printing technology, and material specifications.

3D Printer	Manufacturer	Technology	Material	Tensile Strength (MPa)
Objet30 Prime	Stratasys Ltd., Minneapolis, MN, USA	MJ ^1^	MED610 SUP705 ^5^	50–65
Form 3	Formlabs Inc., Somerville, MA, USA	SLA ^2^	Dental SG	≥50
Ultimaker 3 Extended	Ultimaker B.V., Utrecht, The Netherlands	FFF ^3^	Nylon680 ProFill™ PVA ^5^	47
Wanhao Duplicator 7 Plus	Wanhao, Jinhua, China	DLP ^4^	Freeprint ortho 405	>75

^1^ Material Jetting; ^2^ Stereolithography; ^3^ Fused filament Fabrication; ^4^ Digital Light Processing; ^5^ water soluble support material.

**Table 3:** Summary of the accuracy (trueness) of RMS values for each printer.

Technology	Printer	Median (Q1 to Q3)	Mean ± SD
MJ ^1^	Objet30 Prime	0.09 (0.08 to 0.09)	0.09 ± 0.01
SLA ^2^	Form 3	0.12 (0.11 to 0.14)	0.12 ± 0.02
FFF ^3^	Ultimaker 3 Ext.	0.17 (0.15 to 0.22)	0.18 ± 0.04
DLP ^4^	Duplicator 7 Plus	0.22 (0.21 to 0.27)	0.25 ± 0.05

^1^ Material Jetting; ^2^ Stereolithography; ^3^ Fused filament Fabrication; ^4^ Digital Light Processing.

**Table 4:** Summary of the described values in the point cloud dataset (mm) for each printer.

Technology	Printer	RMS (Median)	Mean	SD	Median	Minimum	Maximum
MJ	Objet30 Prime	0.09	0.04	0.07	0.03	−1.40	1.95
SLA	Form 3	0.12	0.06	0.11	0.06	−1.59	1.59
FFF	Ultimaker 3 Ext.	0.17	0.03	0.18	0.02	−1.41	1.84
DLP	Duplicator 7 Plus	0.22	0.14	0.20	0.11	−1.04	1.90

**Table 5:** *p*-Values of the Tukey–Kramer post hoc test for the accuracy of 3D printers.

	Form 3	Objet30 Prime	Ultimaker 3 Ext.
Objet30 Prime	0.086		
Ultimaker 3 Ext.	<0.01	<0.01	
Duplicator 7 Plus	<0.01	<0.01	<0.01

## Data Availability

The original contributions presented in the study are included in the article, further inquiries can be directed to the corresponding author.

## Abbreviation

sCAIS	static Computer-Assisted Guided Implant Surgery
3D	Three-dimensional
CBCT	Cone-Beam Computer Tomography
CAD	Computer-Aided Design
CAM	Computer-Aided Manufacturing
AM	Additive Manufacturing
STL	Standard Tessellation Language
MJ	Material Jetting
SLA	Stereolithography
FFF	Fused Filament Fabrication
DLP	Digital Light Processing
IPA	Isopropyl Alcohol
UV	Ultraviolet
RMS	Root Mean Square
SD	Standard Deviation
QR	Quartile Ranges
ANOVA	Analysis of Variance
Q-Q	Quantile-Quantile
